# *Streptomyces fradiae* Mitigates the Impact of Potato Virus Y by Inducing Systemic Resistance in Two Egyptian Potato (*Solanum tuberosum* L.) Cultivars

**DOI:** 10.1007/s00248-024-02437-5

**Published:** 2024-10-17

**Authors:** Fafy A. Mohammed, Samah H. Abu-Hussien, Noha K. El Dougdoug, Neima Koutb, Abdalla S. Korayem

**Affiliations:** 1 Botany Department, Faculty of Women for Arts, Science and Education, Ain Shams University, Cairo, 11757 Egypt; 2 Agricultural Microbiology Department, Faculty of Agriculture, Ain Shams University, Cairo, 11241 Egypt; 3 Department of Botany and Microbiology, Faculty of Science, Benha University, Benha, Egypt; 4 Genetics Department, Faculty of Agriculture, Ain Shams University, Cairo, 11241 Egypt

**Keywords:** Potato virus Y, *Streptomyces fradiae*, Antiviral activity, 16S rRNA, Systemic resistance, Potato cultivars

## Abstract

In this study, the impact of culture media filtrate of QD3 actinobacterial isolate on two potato cultivars, Spunta and Diamond, infected with potato virus Y (PVY) was investigated. Various parameters, including infection percentage, PVY virus infectivity, disease severity scoring, PVY optical density, photosynthetic and defense-related biochemical markers, enzymatic profiling, phenolic compounds, proline content, salicylic acid levels, and growth and yield parameters, were assessed to elucidate the potential of the QD3 actinobacterial isolate culture filtrate in mitigating PVY-induced damage. The physiological and biochemical characteristics of the QD3 actinobacterial isolate, including its salinity tolerance, pH preferences, and metabolic traits, were investigated. Molecular identification via 16S rRNA gene sequencing confirmed its classification as *Streptomyces fradiae* QD3, and it was deposited in GenBank with the gene accession number MN160630. Distinct responses between Spunta and Diamond cultivars, with Spunta displaying greater resistance to PVY infection. Notably, pre-infection foliar application of the QD3 filtrate significantly reduced disease symptoms and virus infection in both cultivars. For post-PVY infection, the QD3 filtrate effectively mitigated disease severity and the PVY optical density. Furthermore, the QD3 filtrate positively influenced photosynthetic pigments, enzymatic antioxidant activities, and key biochemical components associated with plant defense mechanisms. Gas chromatography‒mass spectrometry (GC‒MS) analysis revealed palmitic acid (hexadecanoic acid, methyl ester) and oleic acid (9-octadecanoic acid, methyl ester) as the most prominent compounds, with retention times of 23.23 min and 26.41 min, representing 53.27% and 23.25%, respectively, of the total peak area as primary unsaturated fatty acids and demonstrating antiviral effects against plant viruses. Cytotoxicity assays on normal human skin fibroblasts (HSFs) revealed the safety of QD3 metabolites, with low discernible toxicity at high concentrations, reinforcing their potential as safe and effective interventions. The phytotoxicity results indicate that all the seeds presented high germination rates of approximately 95–98%, suggesting that the treatment conditions had no phytotoxic effect on the *Brassica oleracea* (broccoli) seeds, *Lactuca sativa* (lettuce) seeds, and *Eruca sativa* (arugula or rocket) seeds. Overall, the results of this study suggest that the *S. fradiae* filtrate has promising anti-PVY properties, influencing various physiological, biochemical, and molecular aspects in potato cultivars. These findings provide valuable insights into potential strategies for managing PVY infections in potato crops, emphasizing the importance of *Streptomyces*-derived interventions in enhancing plant health and crop protection.

**Supplementary Information:**

The online version contains supplementary material available at 10.1007/s00248-024-02437-5.

## Introduction

Plant viruses are the second most common cause of plant diseases after fungi and are responsible for major agricultural losses every year. The global economic impact of plant viruses is estimated to be approximately $60 billion annually, with $20 billion attributed to losses in food crops alone. PVY (genus Potyvirus, family Potyviridae) is the most prevalent viral pathogen infecting potatoes and other plants in the Solanaceae family [1]. As one of the most damaging viruses in this family, PVY infects several agriculturally important crops, including potatoes, peppers, tobacco, and tomatoes, leading to substantial declines in yield [2]. Additionally, its efficient transmission occurs through vegetative propagation, seed tubers, and aphid vectors, which facilitate rapid spread [1]. In Egypt, potato (*Solanum tuberosum* L.) is a crucial crop for both national food security and export markets. It ranks second only to tomatoes in terms of total vegetable production and cash crop value within Egyptian horticulture [3]. Potatoes constitute the largest export of vegetables in Egypt, with 70–90% of exports going to the European Union in recent years [4]. PVY infection induces an array of foliar symptoms in potatoes, ranging from mild mosaic patterns to more severe mosaic patterns, leaf necrosis, crinkling, stunting, and leaf drop, depending on the cultivar [5]. These symptoms subsequently lead to degraded quality and reduced yields. While chemical pesticides have been widely used for pest control in agriculture, there are no chemical “viricides” available on the market for directly controlling plant virus diseases. Instead, virus management often relies on indirect methods such as vector control and cultural practices [6]. However, issues with prolonged recovery periods, poor survival of virus-infected plants post-treatment, and inherent toxicity negatively impacting farmers, consumers, and the environment indicate the need for alternative modes of plant virus control [7]. Within the last decade, increasing investigations of biological control approaches using microorganisms or their metabolites have shown promising antiviral efficacy against plant viruses through mechanisms such as induced systemic resistance (ISR) or direct interaction with the virus through antimicrobial compound production [8, 9]. Terrestrial actinobacteria, especially those from the prolific genus *Streptomyces*, have been extensively studied for their ability to produce a diverse array of bioactive secondary metabolites, including antivirals, antifungals, antibacterials, antiprotozoals, antioxidants, antiinflammatories, immunomodulators, and plant growth promoters [9, 10]. Thousands of bioactive small molecules have been discovered from terrestrial *Streptomyces* species, comprising the source of more than two-thirds of the commercially important antibiotics in use today, including antifungals such as nystatin; antibacterials such as streptomycin; and antiviral agents such as blasticidin [11]. *Streptomyces* species isolated from rhizosphere soils have shown particular promise, producing a range of antiviral fatty acids and related compounds. Oleic acid appears especially relevant: exogenous applications to plant tissues can mitigate infections from diverse viruses [12], including PVY, reducing viral RNA replication and disease severity [3]. The prominent production of oleic acid and related polyunsaturated fatty acids such as palmitic [13] and linoleic acid [14] within the metabolic profiles of *Streptomyces* strains provides evidence that these natural metabolites likely contribute to antiviral effects [8]. This briefly introduces the rationale behind exploring these *Streptomyces* metabolites as biological control agents against PVY in potato crops, which are suggested to act as elicitor molecules for the induction of ISR.

The *Streptomyces* culture filtrate shows promise as a biocontrol agent against PVY in potatoes, functioning through direct antiviral activity and enhanced plant defense activation [15]. The current study highlights the antiviral efficacy of marine QD3 actinobacterial secondary metabolites by direct or indirect means, further underscoring their potential for controlling plant viruses. Additional investigations into the spectrum of activity against other agriculturally damaging viruses are still needed to fully characterize antiviral utility in diverse Pathosystems and crop types. Importantly, survey data on the cytotoxicity of marine *Streptomyces* extracts in human cell lines revealed that approximately 75% of strains presented no toxicity, indicating that metabolite mixtures can likely be safely applied as biological control treatments [16, 17]. In light of their extensive bioactive secondary metabolism and promise for ecologically sustainable viral disease suppression, further studies on marine *Streptomyces* strains are warranted. This study aimed to isolate and characterize QD3 actinobacterial isolate from Qarun Lake and evaluate its potential as a biocontrol agent against potato PVY in two potato cultivars grown in Egypt, Diamant, and Spunta. Research has also sought to elucidate the underlying defense mechanisms activated in treated plants, including changes in enzyme activities and defense-related biochemical markers. Additionally, the efficacy of the pre- and post-infection treatments of the culture filtrate was compared to determine the optimal application timing for PVY control.

## Materials and Methods

### Plant Materials

Two potato cultivars that were confirmed to be free of viral infection, Diamond and Spunta varieties, were collected from the brown rot research program at the Agricultural Research Centre (ARC), which is affiliated with the Egyptian Ministry of Agriculture and Land Reclamation, according to the IUCN policy statement for collecting plant materials (https://portals.iucn.org/library/efiles/documents/PP-003-En.pdf and cites.org/eng) in Cairo, Egypt. The potato tubers were transplanted into a custom soil mixture containing a 1:3 ratio (by weight) of sand to clay, which was prepared in 30 cm wide pots to serve as the growth medium.

### PVY Viral Strain Preparation

The PVY subtype N strain (genome deposited under accession number LC515211) was obtained from the Virology Laboratory, Faculty of Agriculture, Ain Shams University, Cairo, Egypt. This viral isolate was maintained and propagated in an infected *Datura metel* host. To prepare the viral inoculum for subsequent potato infection trials, infected Datura leaves were crushed and extracted in 0.1 M phosphate buffer solution at pH 7. The clarified crude sap extract was initially passed through cheesecloth filtration. Primary and tertiary-stage potato leaves were then inoculated with a 10^−1^ dilution of the infectious *Datura* sap. The potato plants inoculated for PVY culture and trials were kept under insect-proof conditions within a greenhouse environment set to 26 ± 1 °C with a 16 h photoperiod [3].

### Actinobacterial Isolate

The QD3 actinobacterial isolate was previously isolated from sediment samples collected from Egypt’s Qarun Lake [15], which is located in El-Fayoum Governorate. Initial isolation was carried out using starch casein agar (SCA) media prepared with 50% seawater and incubated at 37 °C for 7 days [16].

### Phenotypic Characteristics of Qd3 Actinobacterial Isolate

The morphological and culture characteristics and reactions of QD3 actinobacterial isolate to Gram stain were examined via light and scanning electron microscopy (SEM) (JEOL JSM 5200, JEOL Technics Ltd., Japan) after cultivation for 7 days on SCA at 37 °C. Spore-bearing hyphae were also studied via the coverslip method [3].

### Physiological and Biochemical Characteristics of Qd3 Actinobacterial Isolate

The biochemical characterization of the QD3 actinobacterial isolate was performed via various tests. These included melanoid pigment production, carbon source utilization, salt tolerance, and enzyme production tests (amylase, lipase, gelatinase, catalase, and urease). Additionally, nitrate reduction, tyrosine degradation, and xanthine degradation tests were conducted. These biochemical tests were carried out following standard protocols [15].

### Molecular Identification of QD3 Actinobacterial Isolate Via 16S rRNA Gene Analysis

For molecular studies, QD3 actinobacterial isolate was identified at the species level via 16S rRNA sequence analysis using a universal set of primers which included 27F (5′-AGAGTTTGATCCTGGCTCAG-3′) and 1492R (5′-GGTTACCTTGTTACGACTT-3′) then amplified via AmpliTaq® DNA polymerase. The amplified products were sequenced via the Big Dye Terminator Cycle Sequencing Kit (Applied Biosystems, USA). The obtained sequence was resolved and analyzed via the Basic Local Alignment Search Tool (BLAST) algorithm (http://www.ncbi.nlm.nih.gov) for pairwise alignment. The sequence was submitted to GenBank, and the accession number was obtained. The resulting sequence reads were trimmed, assembled in BioEdit software (v7.0.4), and then aligned with Clustal W (v4.5.1) to identify the variable 16S region. Phylogenetic analysis entailed BLAST searches against the NCBI database to find the 12 most related sequences. This reference sequence set was used to construct a neighbor-joining cladogram in MEGA 11, and the isolate was situated taxonomically via bootstrap resampling (1000x) [16].

### Standard Inoculum

To prepare QD3 actinobacterial inoculum before experiments, 100 mL of starch casein broth was inoculated with a loopful of a 7-day-old QD3 culture. This starter culture was grown by incubation at 37 °C with continuous 180 rpm rotary shaking (Model-PSU 2 T plus (Boeco Co., Germany). Actinobacterial cells were harvested after they reached the late logarithmic expansion phase after 7 days by centrifuging the cultures at 10,000 rpm for 15 min at 4 °C to pellet the cells. The pellets were discarded, and the supernatants were collected, and passed through Whatman No. 1 filter paper [15]. The clarified, bioactive QD3 actinobacterial culture filtrate was subsequently used to treat potato plants in the current PVY resistance induction experiments.

### Experimental Design

The potato cultivation experiments took place at the virology greenhouse facility of the Microbiology Department, Faculty of Agriculture, Ain Shams University, Cairo, Egypt, during the 2022 winter growing season. The potato plants were arranged in a completely randomized block design with five biological replicates per treatment. The irrigation schedules and nutritional inputs followed standard agronomic recommendations from the Egyptian Ministry of Agriculture and Land Reclamation. Four experimental groups of potato plants were established: (1) healthy control plants; (2) PVY-infected plants; (3) QD3 actinobacterial filtrate-pretreated plants, which were induced 24 h before PVY infection; and (4) QD3 actinobacterial filtrate-treated plants, which were induced 24 h after PVY infection. Each treatment included twenty individual potato plants. Leaf samples were harvested at 45 days post-viral infection for photosynthesis, enzymes, and defense-related biomarker analyses. Additionally, tuber yield samples were collected at 90 days and aligned with crop maturity. All experimental guidelines complied with relevant standard protocols from the Agricultural Research Centre (ARC) under the Egyptian Ministry of Agriculture and Land Reclamation [3].

### Determination of Disease Index and Severity

To follow up on the progression of PVY disease symptoms over time, periodic visual inspection of foliage on the infected potato plants was conducted. Observations documenting visible disease signs were recorded at 21 days post-viral inoculation, corresponding to an intermediate time point within the potato growing cycle. Each PVY inoculated plant was individually rated for symptoms on a standardized 0–4 scoring scale as described previously [18]. This system captured symptom variation from 0 = no visible symptoms, 1 = mild foliar mosaic mottling (< 20% leaf area affected), 2 = notable mosaic mottling (20–50% leaf area affected), 3 = severe mosaic mottling, stunting, deformity (> 50% leaf area affected), to the maximum score of 4 = extreme mosaic, malformation, necrosis, and plant death. The disease severity (DS) values were calculated at 7, 14, and 21 days after viral inoculation via the following formula [19]:

$$\mathrm{Disease}\;\mathrm{severity}=\frac{\sum\left(\mathrm{disease}\;\mathrm{degree}\times\mathrm{plant}\;\mathrm{numbers}\;\mathrm{in}\;\mathrm{each}\;\mathrm{grade}\right)}{\mathrm{Total}\;\mathrm{number}\;\mathrm{of}\;\mathrm{plants}\times\mathrm{highest}\;\mathrm{disease}\;\mathrm{degree}}$$

### Detection of PVY Virus Infectivity and Quantification

The percentage of PVY virus infectivity was calculated as the number of infected plants per total inoculated plants × 100. A double-antibody sandwich enzyme-linked immunosorbent assay (DAS-ELISA) was used to identify PVY infection and determine the virus optical density (OD) in potato leaves at 7, 14, and 21 days, as described by Adam and Clark. DAS-ELISA was performed to detect and quantify PVY in potato leaf samples. Microtiter plates were coated with PVY-specific capture antibodies, followed by the addition of plant extracts. After incubation and washing, enzyme-conjugated detection antibodies were applied. The substrate p-nitrophenyl phosphate was added, and the resulting color change was measured spectrophotometrically at 405 nm. The samples were considered positive for PVY if OD values exceeded twice the mean OD of healthy controls. The DAS-ELISA procedure was carried out in triplicate for each treatment, with specificity tailored to the respective cultivar [20].

### Assessment of Photosynthesis and Defense-Related Biochemical Markers

#### Photosynthetic Pigments

Chlorophyll a, b, and total carotenoids were extracted from 1 g of fresh leaves via acetone (100%), and the absorbances at 663.2, 644.8, and 470 nm were measured spectrophotometrically via a Spectronic 20D spectrophotometer (Thermo Electron) [21].

#### Total Soluble Phenols

Fresh leaves (1g) were ground in 80% ethanol and kept in a dark container at 0 °C for 24 h. The Folin‒Ciocalteu method was employed. The absorbance was measured at 725 nm, and the concentrations were calculated via a catechol standard curve, expressed as mg catechol equivalents per g fresh weight (FW) [22].

#### Free Proline

One gram of fresh leaves was homogenized in 3% aqueous sulfosalicylic acid. The acid-ninhydrin method was used, and the absorbance was read at 520 nm. The proline concentration (mg/g FW) was determined via a standard curve, with toluene as the blank [18].

#### Free Salicylic Acid

Leaf powder (1g), frozen in liquid nitrogen, was extracted with 10 mL of distilled water and centrifuged at 10,000 rpm for 10 min. The free salicylic acid in the supernatant was reacted with ferric chloride to form a violet complex, which was measured at 540 nm. The salicylic acid concentration (mg/g FW) was calculated via a standard curve [23, 24].

### Enzymatic Profiling of Treated Potato Plants

Apical buds from treated potato plants were used for enzyme extraction. A sample (2g) was homogenized in 10 mL of phosphate buffer (pH 6.8) and centrifuged at 2000 rpm for 20 min at 2 °C. The resulting clear supernatant was used for enzyme activity measurements. Peroxidase (POX) and superoxide dismutase (SOD) activities were determined spectrophotometrically via a UV spectrophotometer (Spectronic 20D, Thermo Electron). POX activity was measured at 420 nm, while SOD activity was assessed at 325 nm. Peroxidase (POX) activity was determined via the use of guaiacol as a substrate. The reaction mixture contained 50 mM phosphate buffer (pH 6.8), 20 mM guaiacol, and 20 mM H_2_O_2_. The increase in absorbance at 470 nm due to guaiacol oxidation was measured over 1 min. One unit of POX activity was defined as the amount of enzyme that caused a 0.01 increase in absorbance per minute. Superoxide dismutase (SOD) activity was assessed by its ability to inhibit the photochemical reduction of nitroblue tetrazolium (NBT). The reaction mixture included 50 mM phosphate buffer (pH 7.8), 13 mM methionine, 75 μM NBT, 2 μM riboflavin, and 0.1 mM EDTA. One unit of SOD activity was defined as the amount of enzyme required to cause 50% inhibition of NBT reduction at 560 nm [18].

### Yield Parameters of Treated Potato Plants

The yield characteristics of the treated potato plants were assessed by harvesting 3 months after planting. Parameters such as fresh plant weight, leaf area, and the weight and quantity of tubers were determined across all treatment groups [3].

### Gas Chromatography‒Mass Spectrometry (GC‒MS)

Before gas chromatography‒mass spectrometry (GC‒MS) analysis was conducted, the metabolites present in the cell-free supernatant filtrate were extracted via centrifugation at 10,000 rpm for 15 min. The resulting extract was then dehydrated using anhydrous Na_2_SO_4_ and a rotary evaporator, followed by dissolution in methanol. Subsequently, GC–MS analysis was performed to examine the metabolites generated during the growth of the QD3 actinobacterial isolate. The chemical composition of the culture filtrate was determined via a TRACE GC-TSQ mass spectrometer (Thermo Scientific, Austin, TX, USA) with a TG–5MS direct capillary column (30 m × 0.25 mm × 0.25 µm film thickness). The temperature profile of the column oven was initiated at 50 °C and increased by 5 °C/min until reaching 250 °C, where it was maintained for 2 min. The temperature was subsequently raised to the final level of 300 °C at a rate of 30 °C/min, followed by a 2-min holding period. The injector and MS transfer line temperatures were set at 270 and 260 °C, respectively. Helium served as the carrier gas at a consistent flow rate of 1 mL/min. The solvent delay was set to 4 min, and an autosampler AS1300 coupled with GC in split mode automatically injected 1 µL of the diluted sample. Electron ionization (EI) mass spectra were collected at an ionization voltage of 70 eV, covering a *m/z* range of 50–650 in full scan mode. The ion source temperature was maintained at 200 °C. Identification of the components was achieved through comparison of their mass spectra with those present in the WILEY 09 and NIST 14 mass spectral databases [16].

### Phytotoxicity of Qd3 Actinobacterial Filtrate

The phytotoxic effects of QD3 culture filtrate were evaluated using seeds from *Brassica oleracea* (Broccoli) seeds, *Lactuca sativa* (lettuce) seeds, and *Eruca sativa* (arugula or rocket) seeds. For each trial, ten seeds were placed in a petri dish and subjected to daily watering with 3 mL of either a 100 mg/L solution of QD3 culture filtrate, or distilled water as a control. The seeds were kept at room temperature for a week, with the experiment conducted in triplicate.

To determine the phytotoxicity, germination rate was calculated after the 7-day period using the following formula:

1 $$\tt \text{Phytotoxicity of QD}3\text{ filtrate }=\frac{\text{Number of seeds that germinated }}{T\text{otal seeds planted}}*100$$

### Cytotoxicity of the Qd3 Actinobacterial Isolate to Normal Human Skin Fibroblasts (Hsfs)

Cytotoxicity assay was conducted using human skin fibroblast (HSF) cells procured from Nawah Scientific, Inc. (Mokatam, Cairo, Egypt). The cells were cultured in DMEM supplemented with 100 mg/mL streptomycin, 100 units/mL penicillin, and 10% heat-inactivated fetal bovine serum in a humidified 5% (v/v) CO_2_ atmosphere at 37 °C. The evaluation of cell viability was performed through an SRB assay. Aliquots of 100 μL cell suspensions (5 × 10^3^ cells) were placed in 96-well plates and incubated in complete media for 24 h. The cells were subsequently treated with another 100 μL aliquot of media containing QD3 filtrate at various concentrations. After 72 h of exposure, the cells were fixed by replacing the media with 150 μL of 10% TCA and incubated at 4 °C for 1 h. The TCA solution was removed, and the cells were washed five times with distilled water. Next, 70 μL of SRB solution (0.4% w/v) was added, and the plates were incubated in the dark at room temperature for 10 min. The plates were then washed three times with 1% acetic acid and left to air dry overnight. To dissolve the protein-bound SRB stain, 150 μL of Tris (10 mM) was added, and the absorbance was measured at 540 nm via a BMG LABTECH®-FLUOstar Omega microplate reader (Ortenberg, Germany). The cell viability was expressed as the percentage to the control cell viability, which was set at 100% [16].

### Statistical Analysis

All the experiments were performed in five independent replicates. The statistical significance of the differences was determined via the least significant difference (LSD) test at *p* ≤ 0.05 via CoStat statistics software version 6.400 [25].

## Results

### Phenotypic Characteristics of the Qd3 Actinobacterial Isolate

The identification of the QD3 actinobacterial isolate was conducted via established methods. Microscopic examination revealed the presence of aerial mycelia bearing an elongated spiral spore chain (S) comprising more than 35–40 smooth oval-shaped spores, distinct from those originating from verticillate sporophores (Table S1). The characteristic long spore chain and its arrangement were consistent with the features typical of *Streptomyces* spp. The cultural traits of the QD3 actinobacterial isolate demonstrated its robust growth on all tested ISP (International *Streptomyces* Project) media. Notably, the aerial mycelium exhibited various shades of pink across all media, and the substrate mycelium consistently displayed a pink color.

### Physiological and Biochemical Characteristics of the Qd3 Actinobacterial Isolate

Analysis of the physiological parameters shown in Fig. S1 revealed that the QD3 isolate has a sodium chloride tolerance up to 7% and grows optimally at pH 8, within a wider pH range spanning 7–9.5. QD3 tests were negative for melanin pigment production. In carbon utilization assays, the QD3 isolate promoted the positive utilization of various sugars, including L-arabinose, D-xylose, D-glucose, sucrose, rhamnose, raffinose, inositol, and D-galactose, as sole carbon sources. However, it was negative for the ability to use D-fructose, D-mannitol, or sorbitol. Additionally, it can utilize sodium acetate. In terms of biochemical properties, QD3 isolate tests were positive for starch hydrolysis potential, gelatin liquefaction, and catalase enzyme production. The isolate tested negative for urease activity, nitrite reduction, tyrosine degradation, and xanthine degradation. QD3 isolate has a positive effect on nitrate reduction. Taken together, these physiological and biochemical trait assessments aid in taxonomic profiling and identifying the metabolic specialization of this QD3isolate.

### Molecular Identification of the QD3 Isolate Via 16S rRNA

**Fig. 1:**
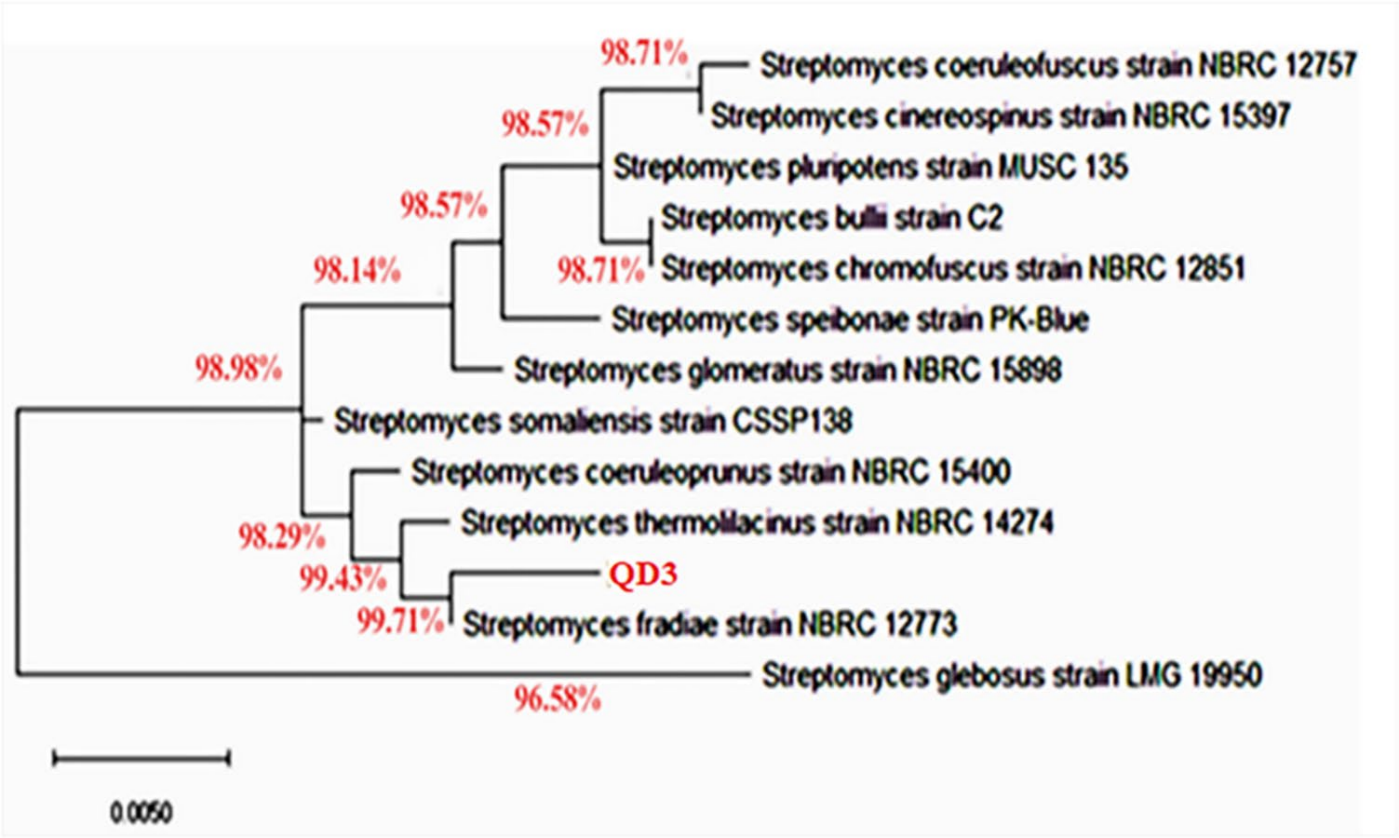
Phylogenetic tree showing the position of the *S. fradiae* QD3 strain with other *Streptomyces* species. The percentage of sequence identity is displayed next to branches. The score bar represents one nucleotide substitution per 1000 nucleotides

According to the phenotypic assessments, the QD3 isolate was classified within the *Streptomyces* genus, as shown in Fig. 1. Further analysis via 16S ribosomal RNA gene sequencing (~ 710 bp amplicon) and BLAST comparisons revealed a 99.71% sequence identity match to *Streptomyces fradiae*. The obtained 16S rRNA sequence was submitted to GenBank as *Streptomyces fradiae* QD3 under gene accession number MN160630. For additional confirmation, a phylogenetic tree was constructed via MEGA X software, which aligns the 16S rRNA data of *S. fradiae* QD3 compared to the top 12 matching *Streptomyces* spp. reference strains from GenBank. The results revealed that the closest phylogenetic position of *S. fradiae* QD3 clustered alongside the *S. fradiae* clade, corroborating species assignment via both genotypic and phenotypic approaches.

### Disease Index and Severity

**Fig. 2:**
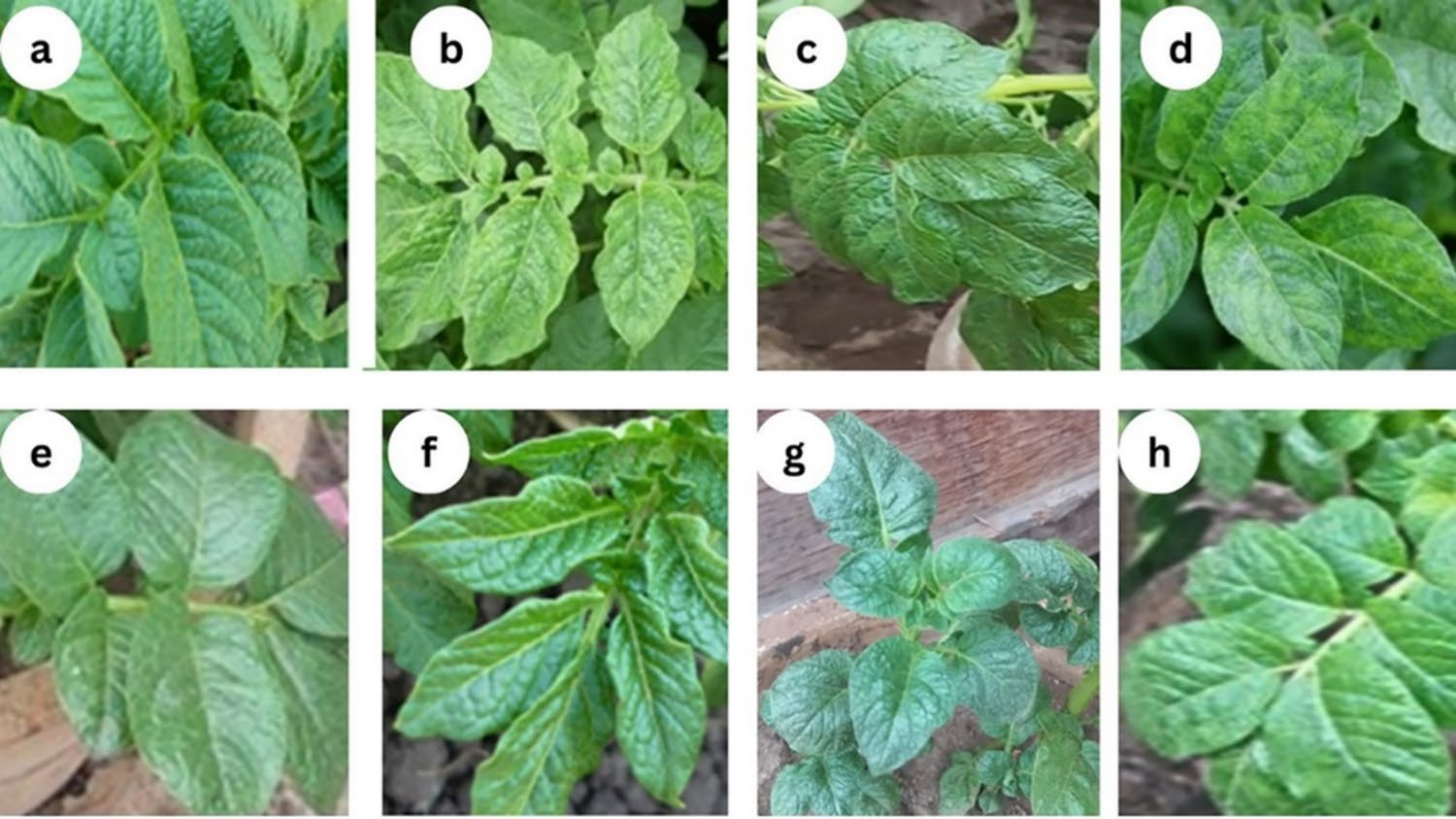
Photographs of Diamond and Spunta potato cultivars after 21 days of infection: **a** healthy Diamond potato plants, **b** PVY-infected plants, **c** PVY-infected plants sprayed with the QD3 filtrate 24 h pre-infection, **d** PVY-infected plants sprayed with the QD3 filtrate 24 h post-infection Diamond cv. Plants, **e** healthy Spunta plants, **f** PVY-infected plants, **g** PVY-infected plants sprayed with metabolites of the QD3 filtrate 24 h pre-infection, **h** PVY-infected plants sprayed with metabolites of the QD3 filtrate 24 h post-infection Spunta plants

The disease index quantifies the severity of PVY infection in Diamond and Spunta potato cultivars. At 21 days post-infection, untreated Diamond plants showed strong disease symptoms (severe mosaic, vein enation, leaf rugosity, venial necrosis, and mottling), while untreated Spunta plants had mild symptoms (mild mosaic, leaf narrow, and leaf rugosity) indicating Diamond cv. is more susceptibility to PVY infection. Treatment with *S. fradiae* QD3 filtrate significantly reduced the disease index in both cultivars, with pre-infection treatment being more effective than post-infection treatment (Table S2 and Fig. 2).

**Fig. 3:**
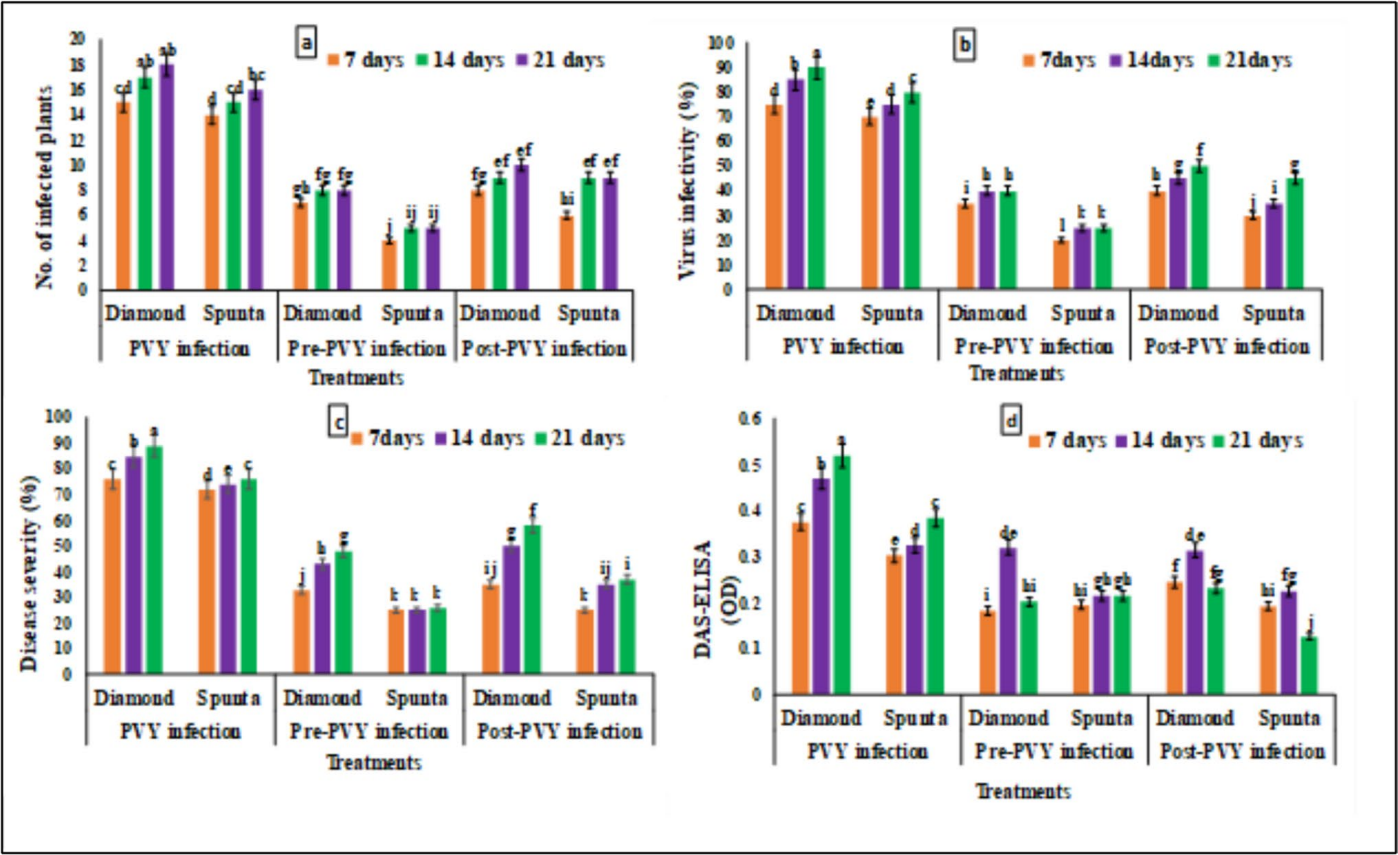
Evaluation of foliar parameters and potato virus Y (PVY) infection in two potato cultivars under different time periods (7, 14, 21 days) with *S. fradiae* QD3 filtrate. **a** No. of infected plants, **b** virus infectivity (%), **c** disease severity (%), **d** DAS-ELISA (OD at 405 nm). The means within each column marked with different letters are significantly different (P < 0.05), and each value represents the mean of five replicates ± SD

The disease severity percentages for the Diamond and Spunta potato cultivars across different treatments and time periods are illustrated in Fig. 3c and Table S2. For PVY infection, Diamond exhibited the highest disease severity, starting at about 76% at 7 days, increasing to 85% at 14 days, and reaching 89% at 21 days. Spunta shows lower severity, with approximately 72% at 7 days, 74% at 14 days, and 76% at 21 days. Pre-PVY infection treatment significantly reduced severity for both cultivars: Diamond showed about 33%, 43%, and 48% severity, while Spunta demonstrated even lower rates at roughly 25%, 25%, and 26% for 7, 14, and 21 days respectively. Post-PVY infection treatment resulted in intermediate severity levels for Diamond and Spunta cultivars. These data suggests that disease severity tends to increase over time especially in untreated or post-infection-treated plants, while pre-infection treatment appears to stabilize severity levels more effectively.

### PVY Virus Infectivity

In the same manner, virus infectivity increased with time recording the highest value of 90% at 21 days for untreated Diamond cv. while the lowest infectivity was for pre-PVY-infected Spunta cv. as presented in Fig. 3b and Table S2. These data indicate that pre-infection treatment is most effective in reducing virus infectivity, with Spunta generally showing lower infectivity rates than Diamond across all conditions.

### Detection of PVY by DAS-ELISA

The presence of PVY in the leaves for all potato cultivars subjected to virus infection was discerned through DAS-ELISA, as delineated in Fig. 3d and Table S2. Untreated Diamond cv. showed the highest DAS-ELISA readings; 0.37 at 7 days, 0.47 at 14 days, and up to 0.52 at 21 days. Spunta exhibited lower values, with approximately 0.30 at 7 days, 0.32 at 14 days, and 0.38 at 21 days. Pre-PVY infection treatment significantly reduces DAS-ELISA optical density for both cultivars. Post-PVY infection treatment results in intermediate DAS-ELISA levels. These results underscore the efficacy of the *S. fradiae* QD3 filtrate in curbing PVY, highlighting its potential as a valuable intervention in managing PVY-infected potato cultivars both before and after infection. Spunta cv. consistently shows lower DAS-ELISA values than Diamond, suggesting lower virus titers.

### Assessment of Photosynthesis and Defense-Related Biochemical Markers

#### Photosynthetic Pigments

For the control treatments, the data in Table 1 presents the contents of photosynthetic pigments in healthy and PVY-infected potato plants of two different cultivars, Spunta cv. and Diamond cv. The measurements included chlorophyll A, chlorophyll B, and carotenoids, with each value accompanied by a standard deviation. For chlorophyll A, in the healthy state (control), Spunta cv. presented a greater chlorophyll A level (4.55 mg/g) than did Diamond cv. (3.75 mg/g); similarly, for chlorophyll B, Spunta cv. presented a greater chlorophyll B level (1.67 mg/g) than did Diamond cv. (1.38 mg/g) in healthy plants. Moreover, the carotenoid content in healthy Spunta cv. plants (0.86 mg/g) was greater than that in Diamond cv. plants (0.75 mg/g).

**Table 1:** Contents of the photosynthetic pigments in healthy and PVY-infected potato plants of both cultivars after 21 days of infection

Variety		Chlorophyll A (mg/g FW)	Chlorophyll B (mg/g FW)	Carotenoids (mg/g FW)
**Spunta cv.**		4.55^a^ ± 0.89	1.67^a^ ± 0.64	0.86^a^ ± 0.21
**Diamond cv.**		3.75^b^ ± 0.91	1.38^b^ ± 0.67	0.75^a^ ± 0.23
**Treatment**				
**Control**		4.9^a^ ± 0.33	2.14^a^ ± 0.13	1.09^a^ ± 0.08
**PVY-infected plants**		2.7^b^ ± 0.44	0.58^b^ ± 0.08	0.53^b^ ± 0.12
**QD3 pre-PVY infection**		4.65^c^ ± 0.49	1.95^c^ ± 0.05	0.89^c^ ± 0.06
**QD3 post-PVY infection**		4.35^d^ ± 0.49	1.45^d^ ± 0.46	0.74^d^ ± 0.05
**Interaction**				
**Spunta cv.**	**Control**	5.2^a^ ± 0.05	2.25^a^ ± 0.05	1.15^a^ ± 0.05
	**PVY-infected plants**	3.1^ g^ ± 0.02	0.65^f^ ± 0.02	0.63^e^ ± 0.02
	**QD3 pre-PVY infection**	5.1^b^ ± 0.05	1.93^ cd^ ± 0.05	0.92^c^ ± 0.05
	**QD3 post-PVY infection**	4.8^c^ ± 0.05	1.86^d^ ± 0.05	0.75^d^ ± 0.05
**Diamond cv.**	**Control**	4.6^d^ ± 0.05	2.03^b^ ± 0.05	1.02^b^ ± 0.05
	**PVY-infected plants**	2.3^ h^ ± 0.05	0.51^ g^ ± 0.05	0.42^f^ ± 0.05
	**QD3 pre-PVY infection**	4.2^e^ ± 0.05	1.96^bc^ ± 0.05	0.85^c^ ± 0.05
	**QD3 post-PVY infection**	3.9^f^ ± 0.05	1.03^e^ ± 0.05	0.72^d^ ± 0.05

The means within each column marked with different letters are significantly different (*P* ≤ 0.05), and each value represents the mean of five replicates ± SD. *FW* fresh weight

With respect to PVY infection, both cultivars presented a significant reduction in photosynthetic pigments, as illustrated in Table 2. For example, the chlorophyll A content decreased to 2.3 mg/g for the PVY-infected Diamond cultivar and 3.1 mg/g for the PVY-infected Spunta cultivar. Similar trends were observed for chlorophyll B and carotenoids, indicating a negative impact on photosynthetic pigment levels due to PVY infection. Compared with the application of the PVY-infected plants, the application of the *S. fradiae* QD3 filtrate before PVY infection resulted in a notable increase in photosynthetic pigments. For example, chlorophyll A increased from 2.3 to 4.2 mg/g in Diamond cv. and from 3.1 to 5.1 mg/g in Spunta cv. Similarly, post-PVY infection treatment with the *S. fradiae* QD3 filtrate led to an improvement in photosynthetic pigments. Chlorophyll A increased from 2.3 to 3.9 mg/g in Diamond cv. and from 3.1 to 4.8 mg/g in Spunta cv.

**Table 2:** Enzymatic activities in healthy and PVY-infected potato plants of both cultivars after 21 days of infection

Variety		POX activity (U/g FW)	SOD activity (U/g FW)
**Spunta cv.**		0.12^a^ ± 0.05	0.29^a^ ± 0.12
**Diamond cv.**		0.11^a^ ± 0.06	0.16^b^ ± 0.06
**Treatment**			
**Control**		0.07^b^ ± 0.04	0.12^b^ ± 0.06
**PVY-infected plants**		0.1^ab^ ± 0.05	0.18^b^ ± 0.06
**QD3 pre-PVY infection**		0.16^a^ ± 0.05	0.32^a^ ± 0.12
**QD3 post-PVY infection**		0.12^ab^ ± 0.05	0.27^a^ ± 0.1
**Interaction**			
**Spunta cv.**	**Control**	0.08^ab^ ± 0.05	0.15^bc^ ± 0.05
	**PVY-infected plants**	0.12^ab^ ± 0.05	0.22^b^ ± 0.05
	**QD3 pre-PVY infection**	0.15^a^ ± 0.05	0.42^a^ ± 0.05
	**QD3 post-PVY infection**	0.11^ab^ ± 0.05	0.35^a^ ± 0.05
**Diamond cv.**	**Control**	0.05^b^ ± 0.02	0.09^c^ ± 0.05
	**PVY-infected plants**	0.08^ab^ ± 0.05	0.14^bc^ ± 0.05
	**QD3 pre-PVY infection**	0.16^a^ ± 0.05	0.21^b^ ± 0.05
	**QD3 post-PVY infection**	0.13^ab^ ± 0.05	0.19^b^ ± 0.05

The means within each column marked with different letters are significantly different (*P* ≤ 0.05), and each value represents the mean of five replicates ± SD. *FW* fresh weight

For the interaction between potato cultivar and treatment, the data in Table 2 revealed that in both cultivars, the QD3 filtrate treatments (pre- and post-PVY infection) consistently resulted in higher levels of chlorophyll A, chlorophyll B, and carotenoids than did the PVY-infected plants did, indicating a potential mitigating effect of the *S. fradiae* QD3 filtrate on PVY-induced damage. In summary, the data suggest that PVY infection has a detrimental effect on the photosynthetic pigments in both potato cultivars, and the application of the *S. fradiae* QD3 filtrate, especially before PVY infection, appears to alleviate these negative effects, potentially contributing to enhanced plant health and resilience against PVY.

#### Enzymatic Profiling of Treated Potato Plants

The data in Table 2 presents the peroxidase (POX) and superoxide dismutase (SOD) enzymatic activities in two potato cultivars, Spunta and Diamond, under various treatments. In terms of POX activity, Spunta consistently presented higher levels than did Diamond across all the treatments. Specifically, in the control, Spunta presented a POX activity of 0.12 U/g, whereas Diamond presented 0.11 U/g. Notably, the application of the QD3 filtrate, both before and after PVY infection, led to an increase in POX activity. In the QD3 pre-PVY infection treatment, Spunta presented the highest POX activity of 0.15 U/g, whereas Diamond presented 0.16 U/g. In terms of SOD activity, Spunta consistently presented higher values than did Diamond across treatments. Specifically, in the control, Spunta had a SOD activity of 0.29 U/g, whereas Diamond had 0.16 U/g. The application of the *S. fradiae* QD3 filtrate, especially in the pre-PVY infection treatment, resulted in elevated SOD activity in both cultivars. These findings underscore the differential responses of Spunta and Diamond to the treatments, with Spunta generally exhibiting higher enzymatic antioxidant activities, indicating the potential of the *S. fradiae* QD3 filtrate in enhancing the defense mechanisms against PVY infection. The interactive comparison indicated that the QD3 pre-PVY infection treatment consistently led to elevated POX and SOD activities in both Spunta and Diamond plants, highlighting its potential for enhancing the antioxidant enzyme response compared with that of the control and PVY-infected plants. Compared with Diamond, Spunta generally presented higher enzyme activities, emphasizing the cultivar-specific responses to the treatments. Overall, the data suggest that the QD3 pre-PVY infection treatment positively influenced the enzymatic antioxidant activities of both cultivars, indicating enhanced defense mechanisms against PVY infection.

#### Phenolic Compounds, Proline Content, and Salicylic Acid

**Fig. 4:**
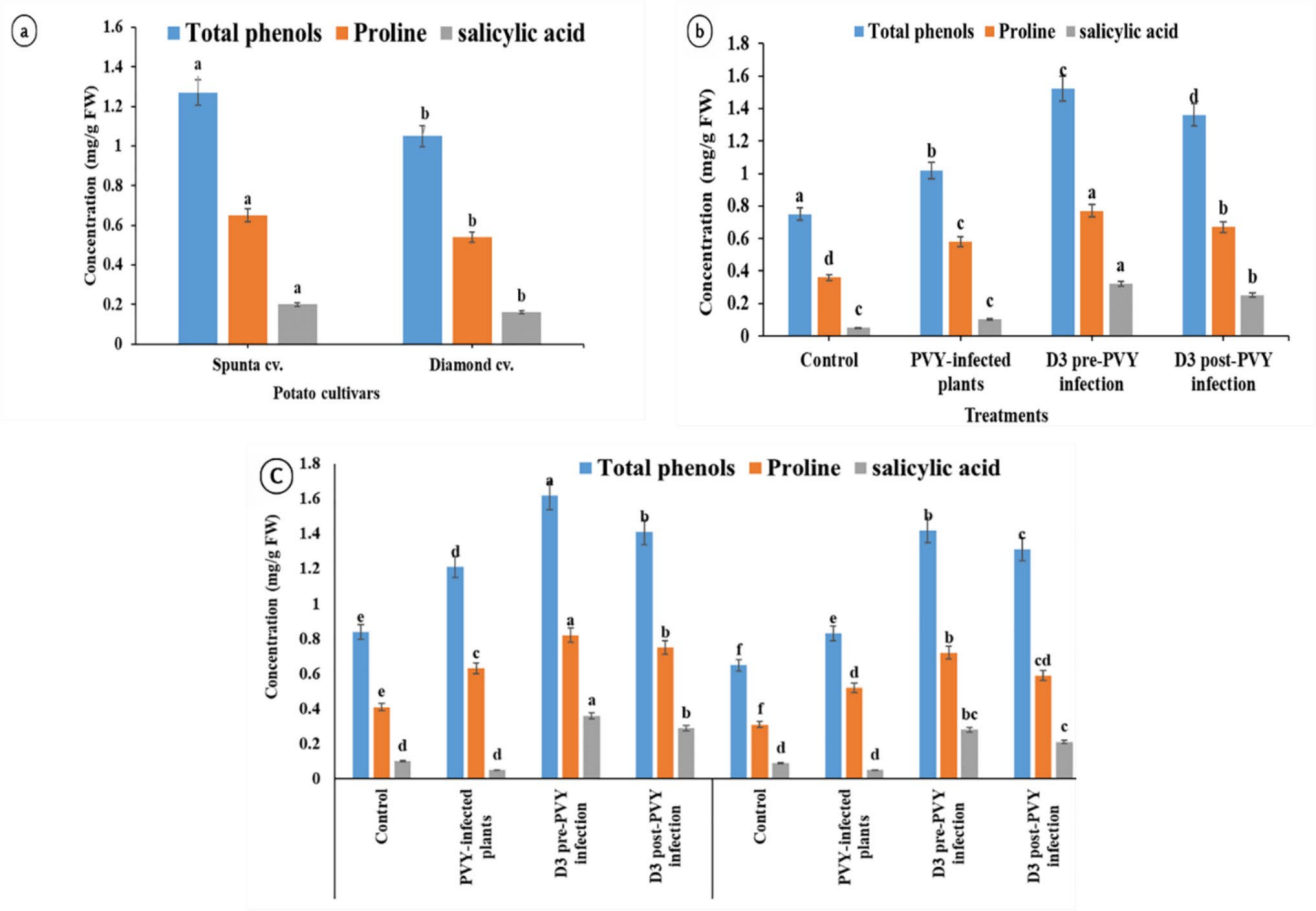
Systemic resistance indices in healthy and PVY-infected potato plants of both cultivars after 45 days of post-infection. **a** Main effects of two cultivars, Spunta and Diamond. **b** Main effects of the four treatments. **c** Interaction between treatment and cultivars. The means within each column marked with different letters are significantly different (*P* < 0.05), and each value represents the mean of five replicates ± SD. FW fresh weight

The data in Fig. 4 provide insights into the levels of total phenols, proline, and salicylic acid in Spunta and Diamond potato cultivars under different treatments. In terms of total phenols, the QD3 pre-PVY infection treatment presented the highest values for both Spunta (1.52 mg/g) and Diamond (1.42 mg/g), with Spunta generally exhibiting relatively high levels. The highest proline content was detected in the QD3 pre-PVY infection treatment, with Spunta (0.77 mg/g) outperforming Diamond (0.72 mg/g). Salicylic acid levels were highest in the QD3 pre-PVY infection treatment for both cultivars, with Spunta (0.36 µg/g) showing higher values than Diamond (0.28 mg/g). The interactive analysis indicated that QD3 pre-PVY infection consistently led to elevated levels of total phenols, proline, and salicylic acid in both Spunta and Diamond, highlighting the potential of this treatment to increase the levels of these biochemical components associated with plant defense mechanisms. Overall, the data highlight the significant impact of the QD3 pre-PVY infection treatment on the accumulation of these key compounds, emphasizing its potential role in fortifying plants against PVY infection.

#### Yield Parameters of Treated Potato Plants

**Fig. 5:**
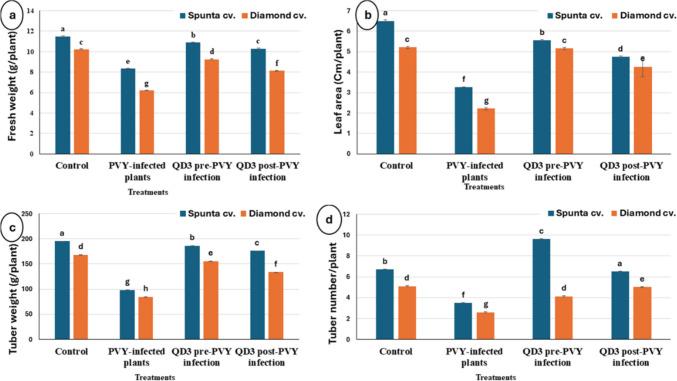
Growth and yield parameters of healthy and PVY-infected potato plants of both cultivars after 90 days of cultivation. **a** Fresh weight (g/plant). **b** Leaf area (cm/plant). **c** Tuber weight (g/plant). **d** Tuber number/plant

The data in Fig. 5 presents the effects of different treatments on various growth parameters in Spunta and Diamond potato cultivars. In the control treatment, Spunta presented a fresh weight of 10.91 g, whereas Diamond presented a slightly lower fresh weight of 10.32 g. The QD3 pre-PVY infection treatment resulted in the highest fresh weight for Spunta (10.08 g) and Diamond (9.25 g). For the leaf area (cm/plant): In the control treatment, Spunta had a greater leaf area (5.86 cm/plant) than did Diamond (5.21 cm/plant). The QD3 pre-PVY infection treatment resulted in a slightly reduced leaf area for both Spunta (5.36 cm/plant) and Diamond (5.16 cm/plant) plants. For tuber weight (g/plant): In the control treatment, Spunta had a greater tuber weight (181.82 g) than did Diamond (168.31 g). The QD3 pre-PVY infection treatment resulted in the highest tuber weights for Spunta (170.96 g) and Diamond (155.61 g). For the number of tubers/plant, in the control treatment, Spunta had a greater number of tubers per plant (5.9 number/plant) than did Diamond (5.1 number/plant). The QD3 post-PVY infection treatment presented the highest tuber number for Spunta (9.6 number/plant) and Diamond (4.12 number/plant). The interactive comparison indicated that the QD3 pre-PVY infection treatment resulted in the highest fresh weight, leaf area, tuber weight, and tuber number for both Spunta and Diamond. Overall, the QD3 pre-PVY infection treatment consistently had positive effects on growth parameters, indicating its potential to increase plant performance even in the presence of PVY infection.

The means within each column marked with different letters are significantly different (*P* ≤ 0.05), and each value represents the mean of five replicates ± SD.

#### Gas Chromatography‒Mass Spectrometry (GC‒MS)

Metabolic profiling of the *S. fradiae* QD3 strains via GC‒MS revealed the production of several fatty acids with potential antiviral activity. As shown in Table 3 and Table S3, the most prominent peak was palmitic acid (hexadecanoic acid, methyl ester), with a retention time of 23.23 min, which represented 53.27% of the total peak area. 6,9-Octadecadienoic acid (oleic acid) was also detected at 26.41 min, at 23.75%. Additional relevant fatty acids included tetradecenoic acids (26.88 min, 4.64% peak area) and 11-octadecatrienoic acid (26.52 min, 3.11%). Together, the presence of these unsaturated and saturated medium-chain fatty acids provides encouraging evidence that antiviral metabolites are produced at appreciable levels by the *Streptomyces* strain under investigation.

**Table 3:**
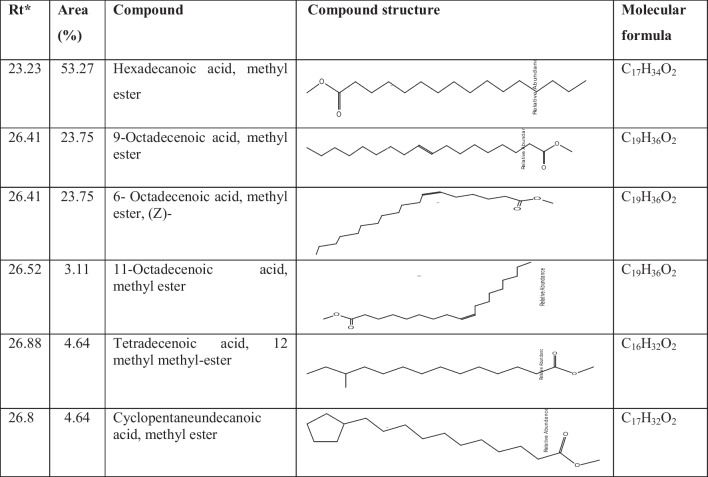
The prominent active metabolites produced by *S. fradiae* QD3 according to GC–MS analysis

Rt* retention time

#### Cytotoxicity of the S. Fradiae Qd3 Strain to Normal Human Skin Fibroblasts (Hsfs)

**Fig. 6:**
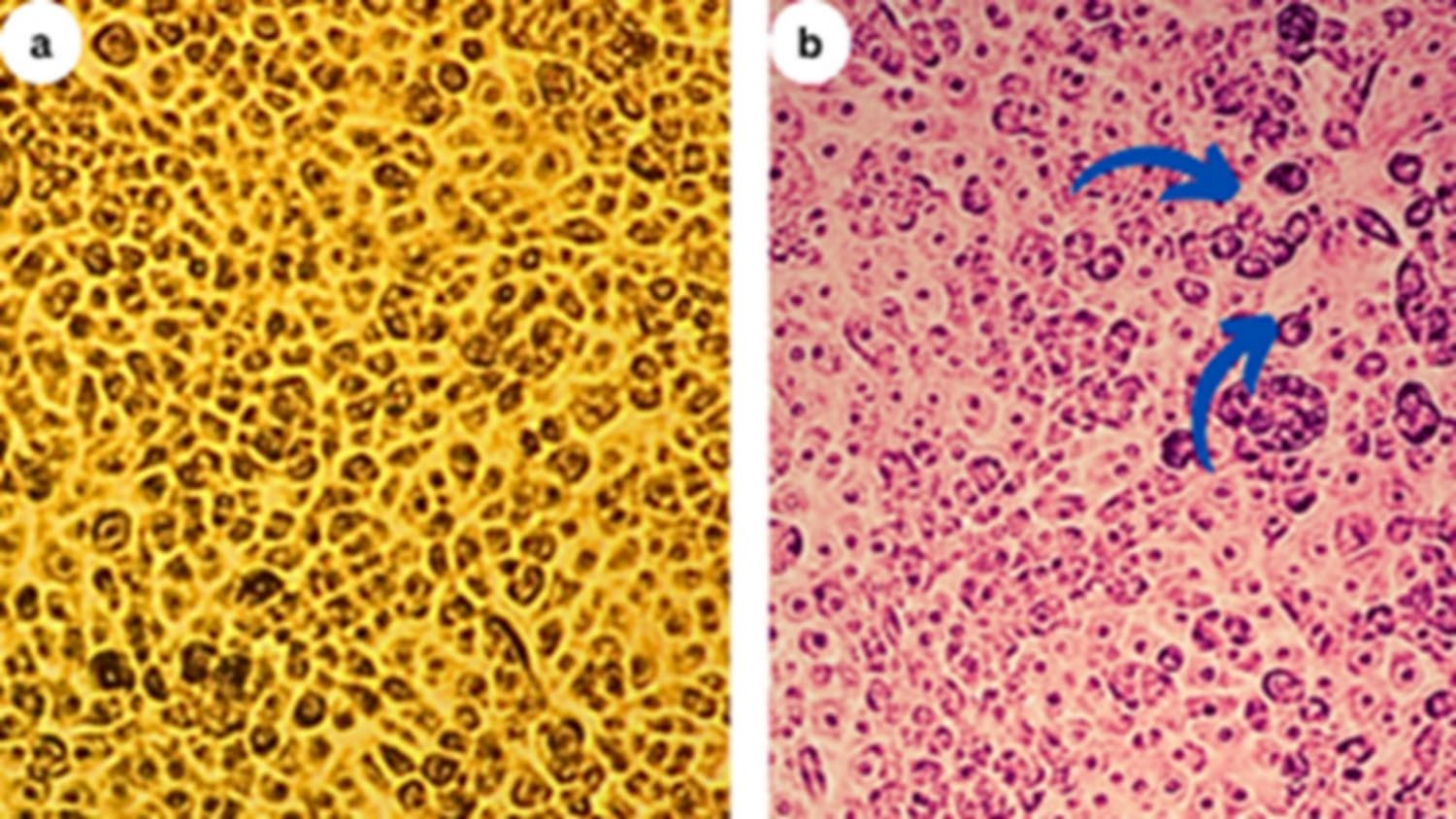
Cytotoxicity of metabolites produced by *S. fradiae* QD3 strain in normal HSF cells maintained in DMEM supplemented with 100 mg/mL streptomycin, 100 units/mL penicillin, and 10% heat-inactivated fetal bovine serum in a humidified 5% (*v*/*v*) CO_2_ atmosphere at 37 °C. **a** Control treatment illustrates normal adherent cells. **b** The cytotoxicity of the *S. fradiae* QD3 metabolites was 92.4% at 100 µg/mL, with few damaged cells, as illustrated by a reduction in cell adhesion

To evaluate potential cytotoxicity, the effects of *S. fradiae* QD3 metabolites across a concentration range from 0 to 100 μg/mL in normal human skin fibroblast (HSF) cells were assessed via an MTT cell proliferation assay. The results revealed no overt toxicity at any tested concentration compared with the untreated controls. Even at the highest dose of 100 μg/mL, HSF viability remained at 99.3%, whereas it reached 92.4% at 100 μg/mL. The calculated half-maximal inhibitory concentration (IC50) value exceeded 100 μg/mL, further indicating negligible toxicity. Additionally, microscopic inspection, as shown in Fig. 6, revealed no discernible differences in HSF morphology or density between metabolite-exposed and control cell populations over the full dose range.

#### Phytotoxic Effects of S. fradiae QD3 Strain on *B. oleracea* Seeds and *L. sativa* Seeds

**Fig. 7:**
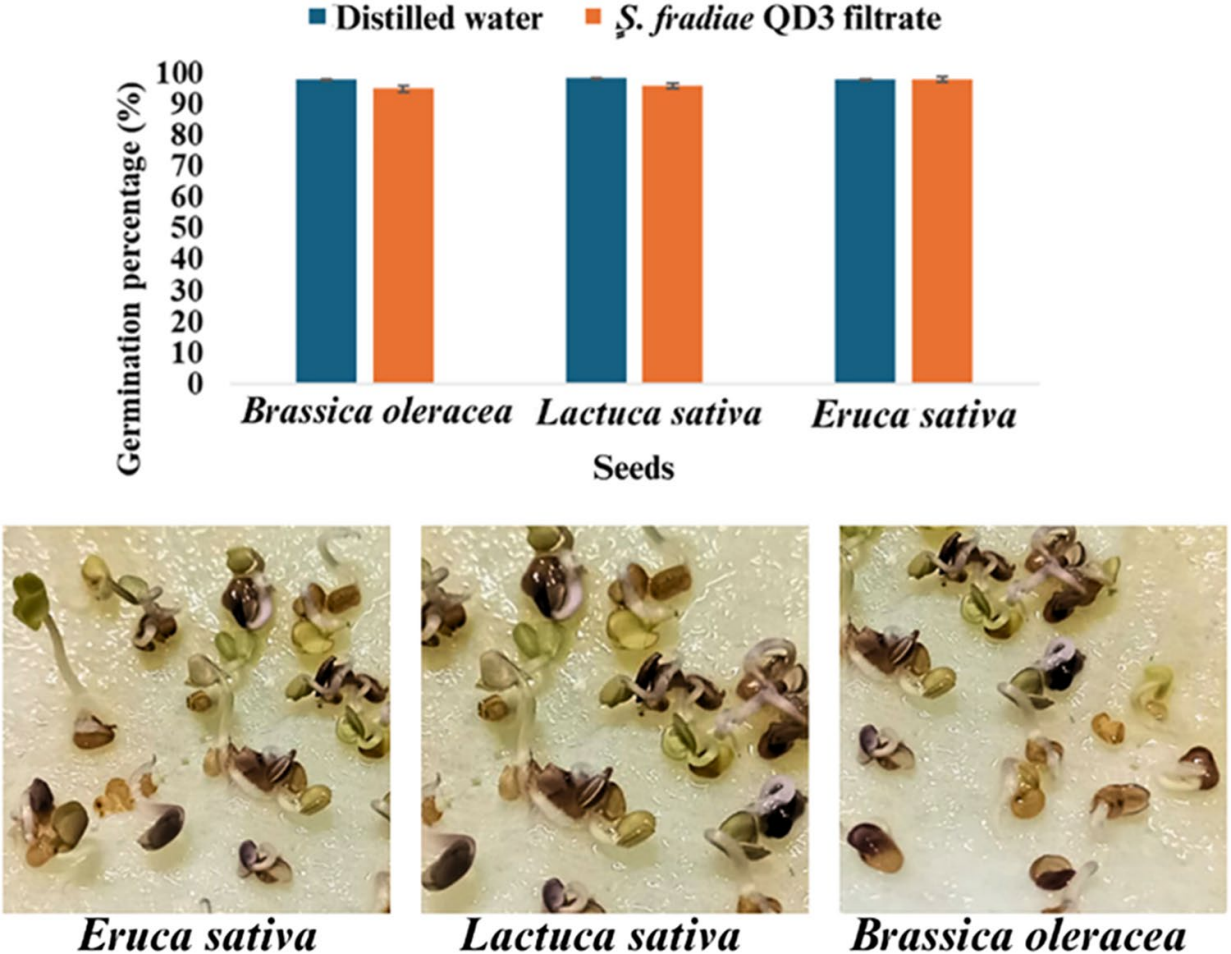
Phytotoxic effects of the *S. fradiae* QD3 culture filtrate on the percentage of germinated seeds (%)

Figure 7 presents a bar graph comparing the germination percentages of *Brassica oleracea* (Broccoli) seeds, *Lactuca sativa* (lettuce) seeds, and *Eruca sativa* (arugula or rocket) seeds via distilled water and the *S. fradiae* QD3 strain culture filtrate. The results indicate that all the seeds exhibited a high germination rate of approximately 95–98%. This high germination rate suggests that the treatment conditions had no phytotoxic effect on any of the seeds used.

## Discussion

In Egypt, PVY poses a significant challenge, causing substantial economic losses. This study explored the use of *Streptomyces fradiae* QD3 culture filtrate to induce ISR in potato plants against PVY infection. The investigation into *S. fradiae* QD3 strain highlights the multifaceted impacts of the actinobacterium on PVY-infected potatoes, which is crucial for managing viral infections and enhancing plant resilience in sustainable agriculture.

This study revealed a reduction in infection percentage, disease severity, and PVY optical density in potato cultivars treated with *S. fradiae* QD3 culture filtrate, which is consistent with previous research on the biocontrol potential of *Streptomyces* sp. against various plant pathogens, including viruses. *Streptomyces* are known for their antagonistic activities, producing secondary metabolites, antibiotics, and enzymes that can suppress viral infections in crops [8].

The differential responses among potato cultivars, such as the greater resistance of the Spunta cultivar than the Diamond cultivar, emphasize the need for tailored biocontrol strategies that consider specific host‒pathogen dynamics. This variability in plant susceptibility is consistent with the literature on the genetic variability in plant responses to viral infections [5].

The concept of ISR is central to this study, where plants, upon exposure to beneficial microorganisms such as *S. fradiae* QD3 strain, activate their defense mechanisms, increasing resistance to subsequent pathogen attacks. *Streptomyces* species are known to induce ISR, and the effectiveness of the *S. fradiae* QD3 filtrate in reducing disease symptoms and virus infection, both pre- and post-PVY infection, suggests its dual functionality as a preventive and responsive measure against PVY [7, 26, 27].

The identification and characterization of QD3 isolate as *S. fradiae* provide insights into its biocontrol potential, notably through the production of diverse secondary metabolites, including antibiotics. The detection of PVY by DAS-ELISA demonstrated a significant reduction in the PVY OD in the treatments with the *S. fradiae* QD3 filtrate, supporting the hypothesis of antiviral activities of *Streptomyces*-derived metabolites [27]. The quantitative detection of the PVY OD via DAS-ELISA further supports the biocontrol potential of *S. fradiae* QD3 strain. The significant reduction in the PVY OD in both the pre- and post-infection treatment groups indicates the ability of the *S. fradiae* filtrate to limit viral proliferation within the plant. This finding aligns with studies demonstrating the antiviral activities of *Streptomyces*-derived metabolites [3]. The precise mechanisms underlying this antiviral activity warrant further investigation, as understanding the mode of action could facilitate the development of targeted and optimized biocontrol strategies [28].

The impact of *S. fradiae* QD3 strain on photosynthetic and defense-related biochemical markers provides insights into the physiological responses of potato plants to PVY infection and treatment. The reduction in photosynthetic pigments upon PVY infection is consistent with the known effects of viral infections on plant photosynthesis [7, 18]. However, the application of the *S. fradiae* QD3 filtrate, especially before PVY infection, led to notable recovery of photosynthetic pigments, indicating potential mitigation of virus-induced damage. Similar trends were observed for enzymatic antioxidant activities, where the *S. fradiae* QD3 filtrate treatments, particularly the pre-PVY infection treatment, increased peroxidase (POX) and superoxide dismutase (SOD) activities. This finding aligns with studies demonstrating the role of *Streptomyces* in enhancing plant antioxidant defenses [3].

The observed increase in total phenol, proline, and salicylic acid levels in response to *S. fradiae* QD3 treatment underscores the activation of defense-related biochemical pathways. These compounds are known to play crucial roles in plant defense against various stresses, including pathogen attacks. The *S. fradiae*–induced accumulation of these biochemical components aligns with the notion that *Streptomyces*-mediated biocontrol involves the activation of systemic defense responses in plants, contributing to enhanced resilience against pathogens [18].

The positive impact of *S. fradiae* QD3 strain on the growth and yield parameters of potato plants, as evidenced by increased fresh weight, leaf area, tuber weight, and tuber number, is consistent with studies demonstrating the growth-promoting effects of beneficial soil microbes. The ability of *Streptomyces* species to increase nutrient uptake, produce growth-promoting hormones, and stimulate root development contributes to improved plant growth and productivity [8].

Gas chromatography‒mass spectrometry (GC‒MS) analysis of *S. fradiae* QD3 metabolites revealed a complex mixture of compounds. While the specific bioactive components responsible for the observed biocontrol effects were not identified in this study, the diversity of metabolites suggests a multifaceted mode of action. Further research focused on isolating and characterizing individual metabolites could provide a more targeted approach to harness the biocontrol potential of *S. fradiae* QD3 strain. Several fatty acids identified via GC–MS analyses have shown promising antiviral effects in previous studies, suggesting that they could have potential for the management of PVY infections. Oleic acid (9-octadecenoic acid) deserves particular attention. Multiple studies have demonstrated the antiviral effects of oleic acid against diverse plant viruses, as it inhibits viral replication through the disruption of virus assembly or the blockade of viral cell-to-cell movement. Specifically, against PVY in potato tissues, exogenous applications of oleic acid reduce viral RNA accumulation, decrease disease severity, and lower rates of viral transmission [3, 12, 13, 29, 30].

The observed antiviral effects of fatty acid disruption of the viral envelope include many viruses, including plant viruses, which have a lipid envelope that is essential for their entry and infection process. Certain fatty acids, particularly those with longer chain lengths and unsaturated bonds, can interact with and disrupt these viral envelopes, rendering the virus particles noninfectious. Interference with viral replication: Some fatty acids have been shown to interfere with various stages of the viral replication cycle, such as viral entry, genome replication, and protein synthesis. They may act by altering cellular membrane fluidity, inhibiting viral enzymes, or modulating host cell signaling pathways involved in viral replication. Induction of plant defense responses: Fatty acids can act as signaling molecules in plants, triggering the activation of defense-related genes and the production of antimicrobial compounds, such as phytoalexins and pathogenesis-related (PR) proteins. These defense responses can confer broad-spectrum resistance against various pathogens, including viruses. Modulation of host‒virus interactions: Fatty acids may influence the interactions between viral proteins and host cellular components, potentially disrupting the hijacking of the host machinery by the virus or altering virus-induced changes in host gene expression or cellular processes. Importantly, the specific mechanisms may vary depending on the viral species, the plant host, and the composition of the fatty acid mixture present in the *S. fradiae* QD3 extract. Further research, including mechanistic studies, gene expression analysis, and molecular docking simulations, would be valuable in elucidating the precise mode(s) of action involved. Additionally, investigating the potential synergistic effects between fatty acids and other bioactive compounds present in the *S. fradiae* QD3 extract, as well as the role of the extract in modulating the overall defense responses of the plant against viral infections, would be beneficial. Oleic acid is consistent with other fatty acids, such as linoleic (9,12-octadecadienoic) acid. Compared with saturated varieties, unsaturated fatty acids tend to have more potent antiviral effects. Another possibly relevant fatty acid, alpha-linolenic acid (9,12,15-octadecatrienoic acid), has also exhibited antiviral properties against plant viruses when applied exogenously to plant tissues, although direct testing against PVY has been more limited. While antiviral efficacy can depend on many factors, such as the timing/method of application, the prominence of oleic acid in the *Streptomyces* metabolic profile is a promising indicator that these bacterial strains could mitigate PVY infections. Potent effects from related polyunsaturated fatty acids also suggest possible synergy among these compounds for improving antiviral defense responses in potato crops. Further planta testing is recommended to confirm and extend these possibilities. The evaluation of *S. fradiae* QD3 metabolite cytotoxicity in normal human skin fibroblasts (HSFs) is crucial for assessing the safety of *S. fradiae* QD3 for potential agricultural applications. The absence of cytotoxic effects on HSF, even at relatively high concentrations, bodes well for the potential use of *S. fradiae* QD3 as a biocontrol agent without posing risks to human health. This aligns with the broader goal of developing environmentally friendly and sustainable agricultural practices.

## Conclusion

In conclusion, the present study underscores the promising role of *S. fradiae* QD3 strain as a biocontrol agent against PVY in potato plants. The observed multifaceted effects, ranging from a significant reduction in disease severity and PVY OD to positive impacts on plant physiology and growth parameters, highlight the potential of *S. fradiae* QD3 in enhancing the resilience of potato crops. Cultivar-specific responses, dual functionality in both preventive and responsive treatments, and the absence of cytotoxic effects on human skin fibroblasts further contribute to its appeal as a sustainable and safe biocontrol option. This study provides critical insights into the complex interactions among *Streptomyces*, the host plant, and the viral pathogen. The identification of QD3 isolate as *S. fradiae*, coupled with the characterization of its metabolites through GC‒MS analysis, offers a foundation for future investigations aimed at revealing the specific bioactive compounds responsible for the observed biocontrol effects. As agriculture faces the challenges of emerging viral plant diseases and the need for sustainable practices, *S. fradiae* QD3 strain has emerged as a promising ally. However, further research, including field trials and a deeper mechanistic understanding of its antiviral activities, is imperative for realizing the full potential of *S. fradiae* QD3 strain as a valuable tool in integrated pest management strategies and sustainable agriculture. The safety of *S. fradiae* QD3 strain and its compatibility with existing agricultural practices make it a candidate for environmentally friendly approaches to combat viral infections in potato crops.

## Supplementary Information

Below is the link to the electronic supplementary material.

Supplementary file1 (PDF 307 KB)

## Data Availability

No datasets were generated or analysed during the current study.
