# Supplementary material for: Streptomyces fradiae Mitigates the Impact of Potato Virus Y by Inducing Systemic Resistance in Two Egyptian Potato (Solanum tuberosum L.) Cultivars
Source: Microb Ecol. 2024 Oct 17;87:131. doi: 10.1007/s00248-024-02437-5 (PMC11486777; doi:10.1007/s00248-024-02437-5)
Supplement: Supplementary file 1 [file 248_2024_2437_MOESM1_ESM.pdf]

Supplementary file

***Streptomyces fradiae* mitigates the impact of Potato Virus Y by inducing systemic resistance in two Egyptian potato (*Solanum tuberosum* L.) cultivars**

Fafy A Mohammed [a], Samah H. Abu-Hussien[b*], Noha K. El Dougdoug [c], Neima Koutb[d], Abdalla S. Korayem[b]

[a] Botany Department, Faculty of Women for Arts, Science and Education, Ain Shams University, Cairo, 11757, Egypt.

[b] Agricultural Microbiology Department, Faculty of Agriculture, Ain Shams University, Cairo, 11241, Egypt

[c] Department of Botany and Microbiology, Faculty of Science, Benha University, Benha, Egypt

[d] Genetics Department, Faculty of Agriculture, Ain Shams University, Cairo, 11241, Egypt

Journal: Microbial Ecology

Corresponding author: Samah H. Abu-Hussien

e-mail: **samah_hashem1@agr.asu.edu.eg**

Table S1: Physiological and biochemical characteristics of the D3 isolate grown for 7 days at 37°C.

Characteristics	Reaction
Physiological characteristics:	
Sodium chloride tolerance	0%–7%
Growth pH	7–9.5
Optimum pH	8
Melanin production	−
Carbon utilization	
L-Arabinose	+
D-xylose	+
D-Glucose	+
Sucrose	+
D-Fructose	-
Rhamnose	+
Raffinose	+
Inositol	+
D-Mannitol	-

D-galactose	+
Sorbitol	-
Sodium acetate	+
Biochemical characteristics	
Starch hydrolysis	+
Gelatin liquefaction	+
Catalase production	+
Urease production	−
Nitrate reduction	+
Nitrite reduction	−
Tyrosine degradation	−
Xanthine degradation	−

positive (+); negative (−)

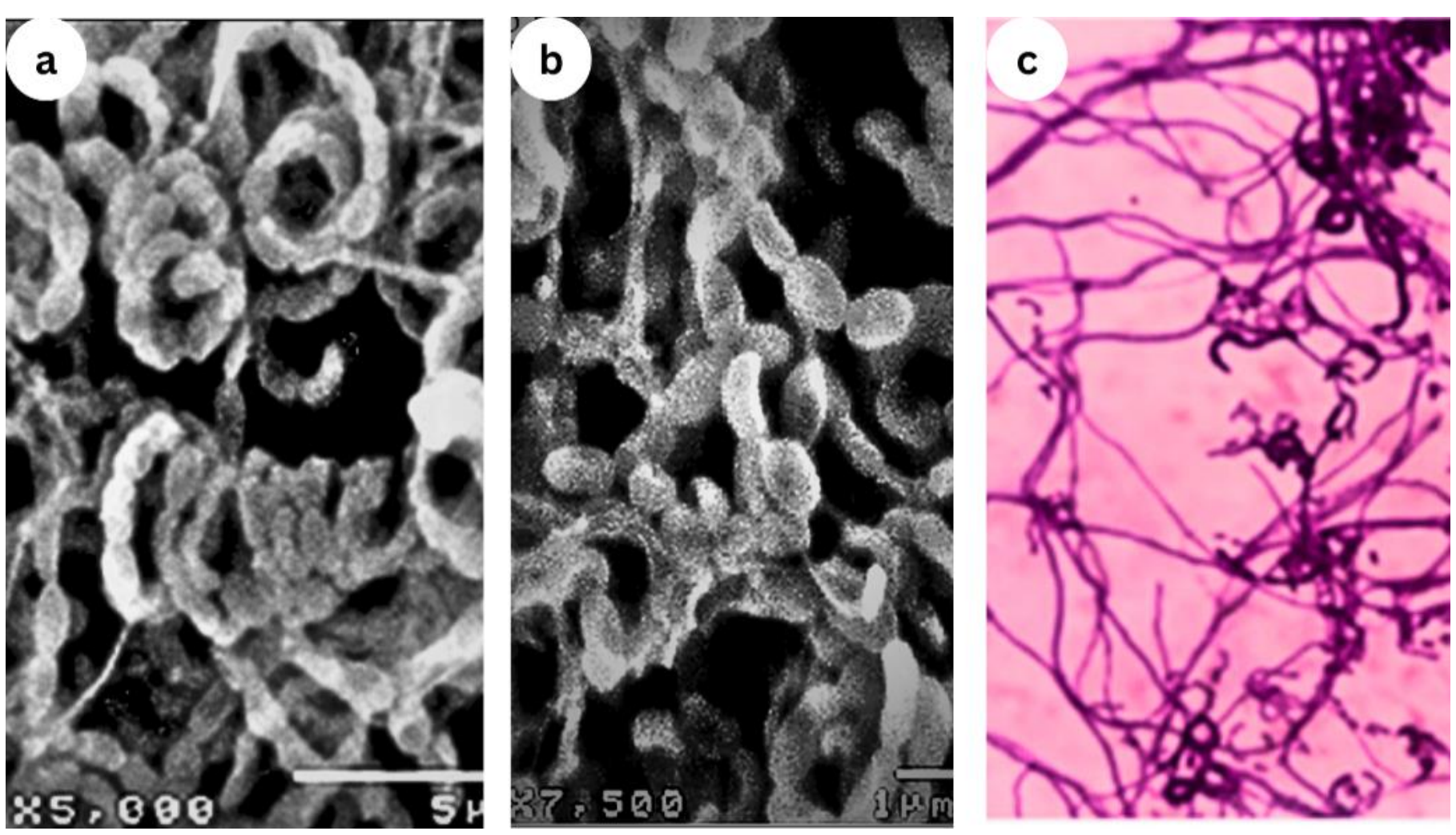

Figure S1: Micrographs of the D3 isolate. Scanning electron micrographs showing (a); the spiral arrangements (5000X) (b); the smooth spore surface(7500X) (c) a light microscope image (1000X) showing aerial mycelium grown on SCA for 7 days at 37°C.

Table S2: Evaluation of foliar parameters and potato virus Y (PVY) infection in two potato cultivars under different foliar treatment conditions with *S. fradiae* QD3 filtrate

**Treatments**	**Potato cultivars**	**No. Infected plants**			**virus infectivity (%)**			**Disease index**			**Disease severity (%)**			**DAS-ELISA OD at 405 nm**		
		**7 days**	**14 days**	**21 days**	**7 days**	**14 days**	**21 days**	**7 days**	**14 days**	**21 days**	**7 days**	**14 days**	**21 days**	**7 days**	**14 days**	**21 days**
PVY infection	Diamond	15/20	17/20	18/20	75	85	90	SM,	SM ,VE,VN LR, Mo		76	85	89	0.37	0.47	0.52
	Spunta	14/20	15/20	16/20	70	75	80	mM,	mM , LN, LR		72	74	76	0.30	0.32	0.38
Pre PVY infection	Diamond	7/20	8/20	8/20	35	40	40	mM	mM, LR, VE		33	43	48	0.18	0.30	0.20
	Spunta	4/20	5/20	5/20	20	25	25	mM	mM , VE		25	25	26	0.19	0.21	0.215
Post PVY infection	Diamond	8/20	9/20	10/20	40	45	50	mM	mM LN,VN, Mo		35	50	58	0.24	0.31	0.23
	Spunta	6/20	9/20	9/20	30	35	45	mM	mM ,LN		25	35	37	0.19	0.22	0.12

SM= Sever mosaic, VE=Vein enation, VN= venial necrosis LR=Leaf rugosity, Mo=Mottling, mM=Mild mosaic, LN= Leaf narrow

Table S3: Spectral analysis of the prominent active metabolites produced by *S. fradiae* by GC-MS analysis.

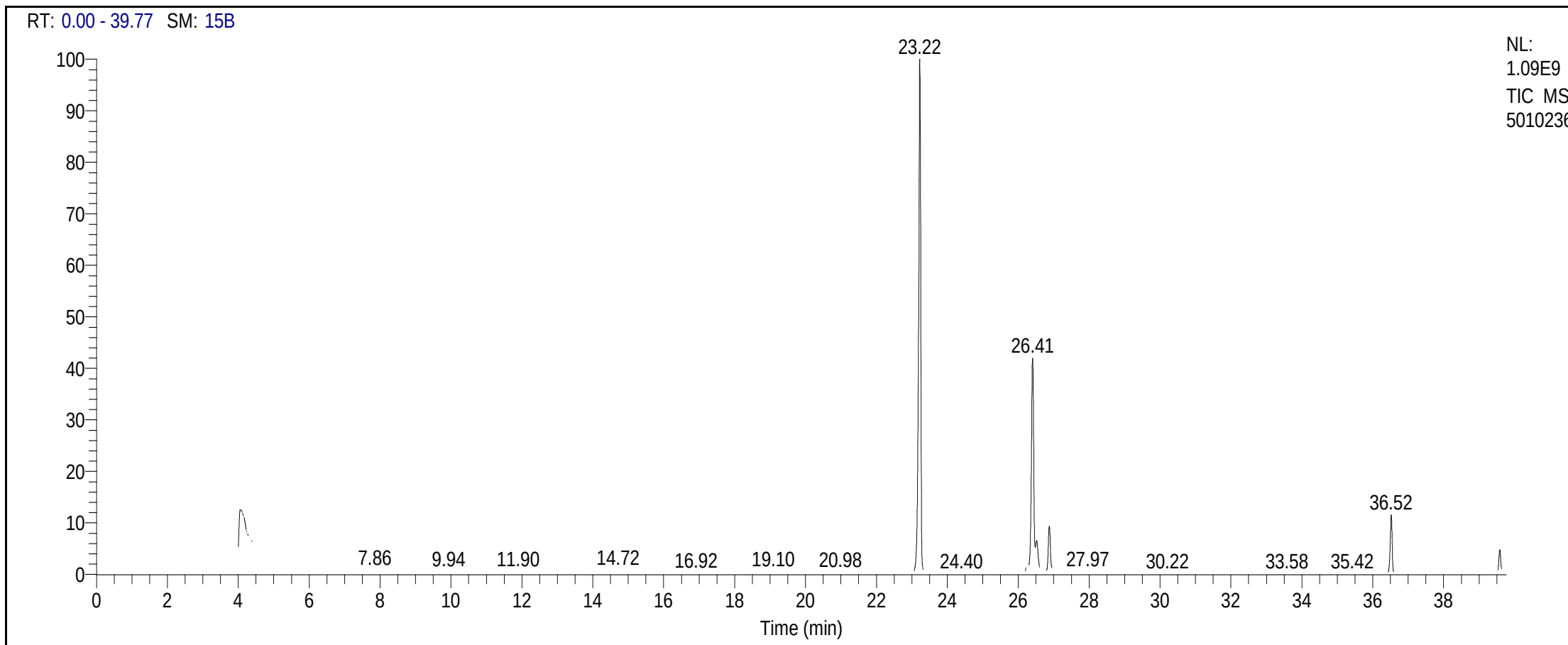

GC-MS Chromatogram displaying the retention times and relative intensities of separated components from D3 isolate filtrate.

Rt*	Area (%)	Compound structure	
23.23	53.27	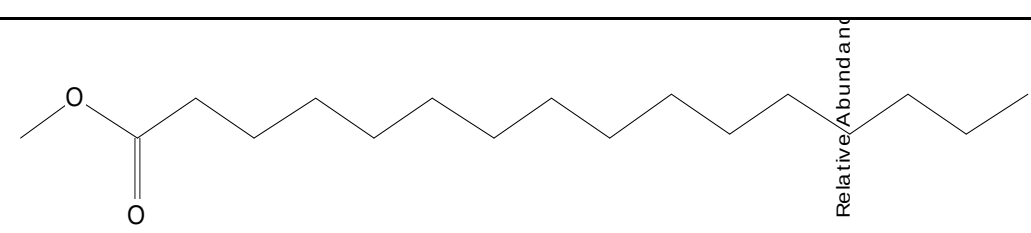	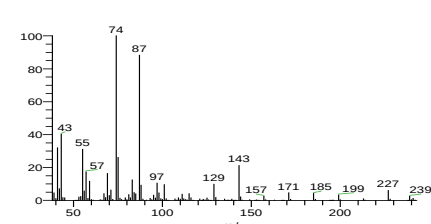
26.41	23.75	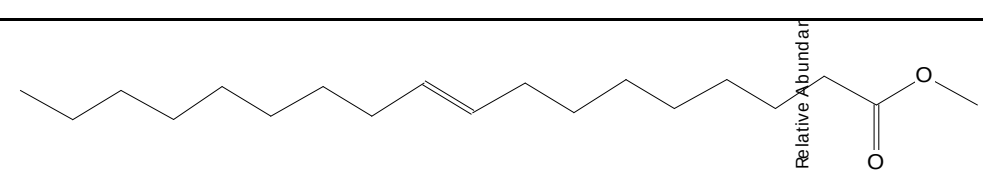	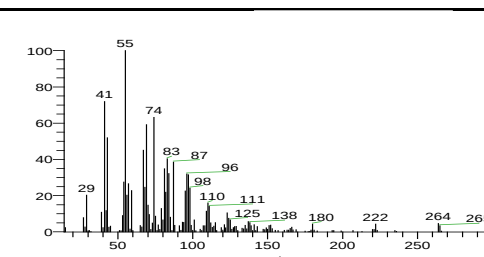
26.41	23.75	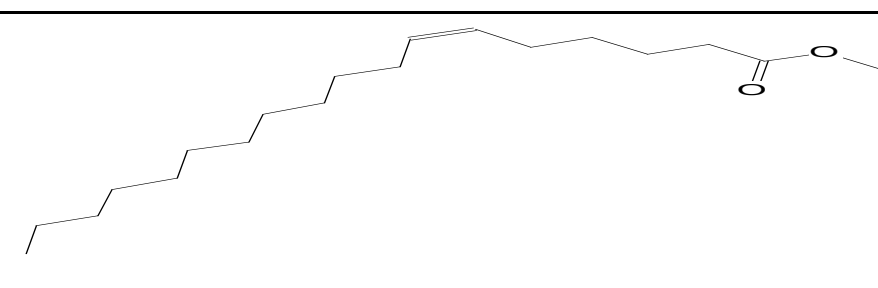	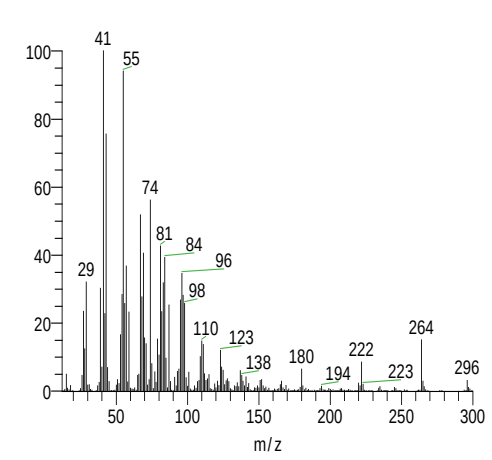
26.52	3.11	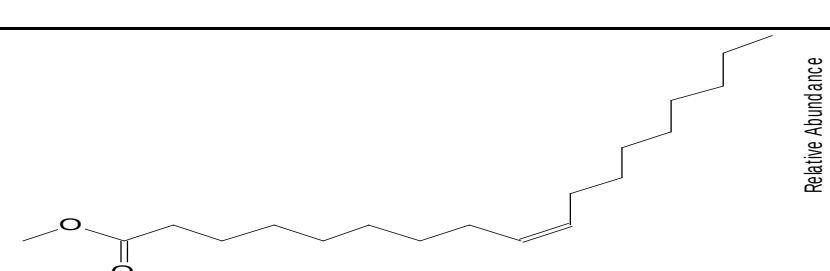	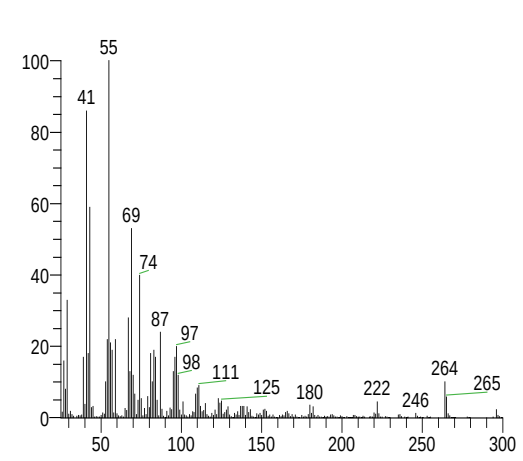

26.88	4.64	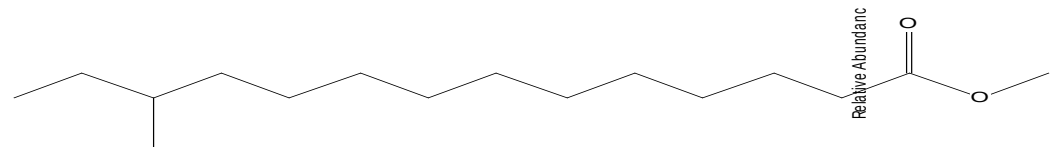	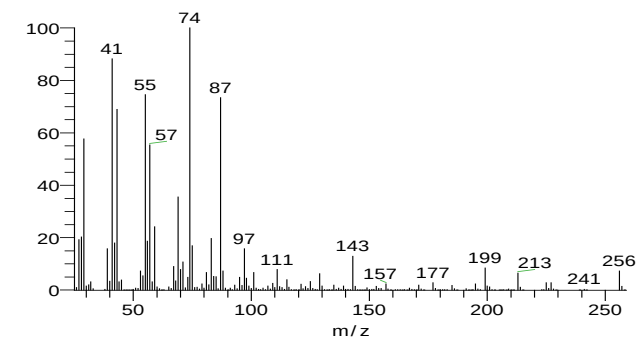
26.8	4.64	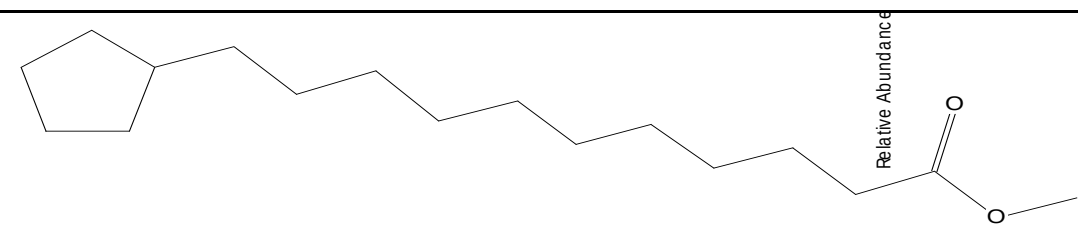	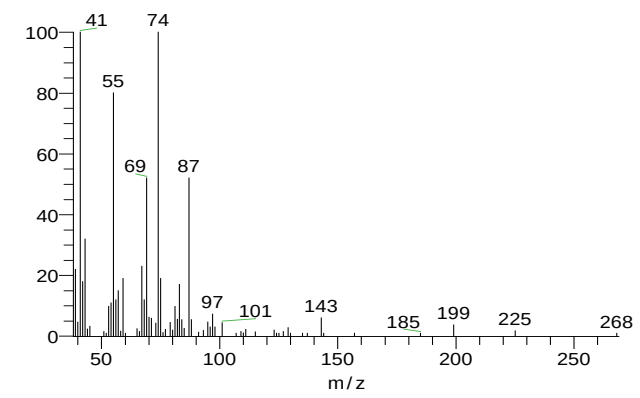
